# Supplementary material for: The longitudinal associations between bone mineral density and appendicular skeletal muscle mass in Chinese community-dwelling middle aged and elderly men
Source: PeerJ. 2021 Jan 19;9:e10753. doi: 10.7717/peerj.10753 (PMC7821753; doi:10.7717/peerj.10753)
Supplement: Supplemental Information 1 [file peerj-09-10753-s001.docx]

**Table S1:**

**Data of low-lean-mass and control group (**$\bar{\boldsymbol{x}}$ **± s) and diagnosis according to SMI <29.9%.**

| **SMI**（%） | **Low-lean-mass group (n = 219)** | **Control group (n = 1125)** | ***P* value** |
| --- | --- | --- | --- |
| **Anthropometric measurement** | | | |
| Age (years) | 58.14 ± 11.41 | 54.24 ± 7.28 | <0.001 |
| Weight (kg) | 76.48 ± 11.52 | 71.70 ± 8.75 | <0.001 |
| Height (cm) | 167.53 ± 6.11 | 170.16 ± 5.66 | <0.001 |
| BMI (kg/m^2^) | 27.19 ± 3.40 | 24.73 ± 2.52 | <0.001 |
| **Body composition** | | | |
| WBTOT_LEAN (g) | 54614.64 ± 7807.66 | 55170.65 ± 6268.27 | 0.050 |
| HEAD_LEAN (g) | 4111.82 ± 355.19 | 4006.32 ± 327.53 | <0.001 |
| LARM_LEAN (g) | 3169.16 ± 534.98 | 3409.12 ± 489.03 | <0.001 |
| RARM_LEAN (g) | 3476.33 ± 565.33 | 3742.36 ± 516.42 | <0.001 |
| TRUNK_LEAN (g) | 27518.74 ± 4335.13 | 26375.11 ± 3320.16 | 0.002 |
| L_LEG_LEAN (g) | 8121.99 ± 1202.23 | 8769.79 ± 1102.77 | <0.001 |
| R_LEG_LEAN (g) | 8216.59 ± 1340.30 | 8867.94 ± 1116.84 | <0.001 |
| **Bone density (g/cm^2^)** | | | |
| T_S_BMD | 0.86 ± 0.12 | 0.88 ± 0.11 | 0.012 |
| L_S_BMD | 0.95 ± 0.14 | 1.00 ± 0.14 | <0.001 |
| PELV_BMD | 1.19 ± 0.16 | 1.23 ± 0.17 | <0.001 |
| LLEG_BMD | 1.12 ± 0.10 | 1.17 ± 0.10 | <0.001 |
| RLEG_BMD | 1.12 ± 0.10 | 1.17 ± 0.10 | <0.001 |
| TOT_BMD | 0.94 ± 0.14 | 0.99 ± 0.15 | 0.003 |
| HIP_BMD | 0.95 ± 0.13 | 0.96 ± 0.13 | 0.422 |
| HIPNECK_BMD | 0.78 ± 0.13 | 0.79 ± 0.12 | 0.181 |
| NECK_BMD | 0.78 ± 0.13 | 0.79 ± 0.12 | 0.082 |

**Notes.**

Data are presented as mean ± SE or number.

BMI, body mass index; HEAD_LEAN, lean mass of head; LARM_LEAN, lean mass of left arm; RARM_LEAN, lean mass of right arm; TRUNK_LEAN, lean mass of trunk; L_LEG_LEAN, lean mass of left leg; R_LEG_LEAN, lean mass of right leg; WBTOT_LEAN, lean mass of whole body; T_S_BMD, thoracic spinal BMD; L_S_BMD, lumbar spinal BMD; PELV_BMD, pelvic BMD; HTOT_BMD, hip BMD; NECK_BMD, femoral neck BMD; LLEG_BMD, left leg BMD; RLEG_BMD, right leg BMD; SMI, skeletal muscle index.
