# Supplementary material for: The longitudinal associations between bone mineral density and appendicular skeletal muscle mass in Chinese community-dwelling middle aged and elderly men
Source: PeerJ. 2021 Jan 19;9:e10753. doi: 10.7717/peerj.10753 (PMC7821753; doi:10.7717/peerj.10753)
Supplement: Supplemental Information 2 [file peerj-09-10753-s002.docx]

**Table S2:**

**Data of low-lean-mass and control group (**$\bar{\boldsymbol{x}}$ **± s) and diagnosis according to ASM/BMI <0.789.**

| **ASM/BMI（**m^2^**）** | **Low-lean-mass group (n = 112)** | **Control group (n = 1232)** | ***P* value** |
| --- | --- | --- | --- |
| **Anthropometric measurement** | | | |
| Age (years) | 60.14 ± 13.01 | 54.40 ± 7.46 | <0.001 |
| Weight (kg) | 71.05 ± 11.83 | 72.60 ± 9.17 | 0.005 |
| Height (cm) | 161.69 ± 4.44 | 170.46 ± 5.36 | <0.001 |
| BMI (kg/m^2^) | 27.10 ± 3.77 | 24.95 ± 2.66 | <0.001 |
| **Body composition** | | | |
| WBTOT_LEAN (g) | 50653.07 ± 7230.61 | 55482.50 ± 6329.14 | <0.001 |
| HEAD_LEAN (g) | 3980.13 ± 339.30 | 4027.46 ± 333.74 | 0.114 |
| LARM_LEAN (g) | 2923.53 ± 468.33 | 3410.61 ± 487.91 | <0.001 |
| RARM_LEAN (g) | 3221.98 ± 535.82 | 3742.38 ± 512.03 | <0.001 |
| TRUNK_LEAN (g) | 25522.85 ± 4247.30 | 26655.88 ± 3443.04 | <0.001 |
| L_LEG_LEAN (g) | 7463.43 ± 1016.53 | 8773.40 ± 1092.04 | <0.001 |
| R_LEG_LEAN (g) | 7541.16 ± 1150.54 | 8872.78 ± 1119.36 | <0.001 |
| **Bone density (g/cm^2^)** | | | |
| L_S_BMD | 0.93 ± 0.13 | 1.00 ± 0.14 | <0.001 |
| PELV_BMD | 1.14 ± 0.15 | 1.23 ± 0.17 | <0.001 |
| LLEG_BMD | 1.09 ± 0.08 | 1.17 ± 0.10 | <0.001 |
| RLEG_BMD | 1.10 ± 0.09 | 1.17 ± 0.10 | <0.001 |
| TOT_BMD | 0.90 ± 0.11 | 0.99 ± 0.15 | <0.001 |
| HIP_BMD | 0.92 ± 0.13 | 0.96 ± 0.13 | 0.008 |
| HIPNECK_BMD | 0.73 ± 0.13 | 0.80 ± 0.12 | <0.001 |
| NECK_BMD | 0.74 ± 0.14 | 0.80 ± 0.12 | <0.001 |

**Notes.**

Data are presented as mean ± SE or number.

ASM/BMI, appendicular skeletal muscle/body mass index; ASMI, appendicular skeletal muscle index; HEAD_LEAN, lean mass of head; LARM_LEAN, lean mass of left arm; RARM_LEAN, lean mass of right arm; TRUNK_LEAN, lean mass of trunk; L_LEG_LEAN, lean mass of left leg; R_LEG_LEAN, lean mass of right leg; WBTOT_LEAN, lean mass of whole body; L_S_BMD, lumbar spinal BMD; PELV_BMD, pelvic BMD; HTOT_BMD, hip BMD; NECK_BMD, femoral neck BMD; LLEG_BMD, left leg BMD; RLEG_BMD, right leg BMD.
