# Supplementary material for: The longitudinal associations between bone mineral density and appendicular skeletal muscle mass in Chinese community-dwelling middle aged and elderly men
Source: PeerJ. 2021 Jan 19;9:e10753. doi: 10.7717/peerj.10753 (PMC7821753; doi:10.7717/peerj.10753)
Supplement: Supplemental Information 3 — (A) Trends of ASMI over time. (B) Trends of SMI over time. (C) Trends of ASM/BMI over time. [file peerj-09-10753-s003.docx]

**Table S3:**

**Associations between low lean mass changes according to SMI and BMDs (n = 1343).**

| **Outcome:*SMI （** % **）** | **Unadjusted**  **β coefficient (95% CI)** | | | **Adjusted***  **β coefficient (95% CI)** | | |
| --- | --- | --- | --- | --- | --- | --- |
|  | **β** | **(95% CI)** | ***P*** | **β** | **(95% CI)** | ***P*** |
| WBTOT_BMD | 0.03 | (0.01, 0.05) | 0.004 | 0.02 | (0.00, 0.04) | 0.034 |
| HEAD_BMD | -0.01 | (-0.01, -0.00) | 0.002 | -0.01 | (-0.01, -0.00) | 0.009 |
| LRIB_BMD | -0.04 | (-0.06, -0.02) | <0.001 | -0.05 | (-0.06, -0.03) | <0.001 |
| RRIB_BMD | -0.02 | (-0.03, -0.00) | 0.009 | -0.02 | (-0.03, -0.00) | 0.014 |
| T_S_BMD | 0.01 | (-0.00, 0.02) | 0.114 | 0.01 | (-0.00, 0.02) | 0.094 |
| L_S_BMD | 0.01 | (0.00, 0.02) | 0.023 | 0.01 | (0.00, 0.02) | 0.023 |
| PELV_BMD | 0 | (-0.01, 0.01) | 0.498 | 0 | (-0.01, 0.01) | 0.348 |
| HTOT_BMD | 0 | (-0.02, 0.03) | 0.871 | 0 | (-0.02, 0.02) | 0.962 |
| NECK_BMD | 0.01 | (-0.01, 0.04) | 0.383 | 0.01 | (-0.02, 0.03) | 0.665 |
| LLEG_BMD | 0.04 | (0.02, 0.05) | <0.001 | 0.02 | (0.01, 0.04) | 0.003 |
| RLEG_BMD | 0.05 | (0.03, 0.06) | <0.001 | 0.03 | (0.02, 0.05) | <0.001 |
| LARM_BMD | 0.07 | (0.04, 0.10) | <0.001 | 0.05 | (0.02, 0.08) | <0.001 |
| RARM_BMD | 0.07 | (0.04, 0.09) | <0.001 | 0.05 | (0.02, 0.07) | <0.001 |

**Notes.**

*Adjusted for age, weight, HbA1c, HDL-C, creatinine, ALT, FT4, diastolic blood pressure, smoking, drinking and exercise.

WBTOT_BMD, mean whole-body BMD; HEAD_BMD, skull BMD; LRIB_BMD, left rib BMD; RRIB_BMD, right rib BMD; T_S_BMD, thoracic spinal BMD; L_S_BMD, lumbar spinal BMD; PELV_BMD, pelvic BMD; HTOT_BMD, hip BMD; NECK_BMD, femoral neck BMD; LLEG_BMD, left leg BMD; RLEG_BMD, right leg BMD; LARM_BMD, left arm BMD; RARM_BMD, right arm BM.
