# Supplementary material for: The longitudinal associations between bone mineral density and appendicular skeletal muscle mass in Chinese community-dwelling middle aged and elderly men
Source: PeerJ. 2021 Jan 19;9:e10753. doi: 10.7717/peerj.10753 (PMC7821753; doi:10.7717/peerj.10753)
Supplement: Supplemental Information 4 [file peerj-09-10753-s004.docx]

**Table S4:**

**Associations between low lean mass changes according to ASM/BMI and BMDs (n = 1343).**

| **Outcome: ASM/BMI**  **（**m^2^**）** | **Unadjusted**  **β coefficient (95% CI)** | | | **Adjusted***  **β coefficient (95% CI)** | | |
| --- | --- | --- | --- | --- | --- | --- |
|  | **β** | **(95% CI)** | ***P*** | **β** | **(95% CI)** | ***P*** |
| WBTOT_BMD | 0.11 | (0.04,0.18) | 0.003 | 0.09 | (0.02,0.16) | 0.01 |
| HEAD_BMD | -0.02 | (-0.03,-0.01) | 0.003 | -0.02 | (-0.03,-0.00) | 0.008 |
| LRIB_BMD | -0.13 | (-0.19, -0.07) | <0.001 | -0.14 | (-0.19, -0.08) | <0.001 |
| RRIB_BMD | -0.05 | (-0.09, -0.01) | 0.014 | -0.05 | (-0.09, -0.01) | 0.02 |
| T_S_BMD | 0.05 | (0.01, 0.09) | 0.018 | 0.05 | (0.01, 0.10) | 0.012 |
| L_S_BMD | 0.07 | (0.03, 0.11) | <0.001 | 0.07 | (0.03, 0.11) | <0.001 |
| PELV_BMD | 0.04 | (0.00, 0.08) | 0.045 | 0.02 | (-0.02, 0.06) | 0.357 |
| HTOT_BMD | 0.04 | (-0.07, 0.16) | 0.444 | 0.04 | (-0.08, 0.15) | 0.531 |
| NECK_BMD | 0.13 | (0.01, 0.25) | 0.033 | 0.11 | (-0.01, 0.23) | 0.065 |
| LLEG_BMD | 0.15 | (0.09, 0.21) | <0.001 | 0.12 | (0.06, 0.18) | <0.001 |
| RLEG_BMD | 0.19 | (0.13, 0.25) | <0.001 | 0.16 | (0.10, 0.22) | <0.001 |
| LARM_BMD | 0.27 | (0.16, 0.38) | <0.001 | 0.22 | (0.12, 0.33) | <0.001 |
| RARM_BMD | 0.26 | (0.17, 0.36) | <0.001 | 0.21 | (0.11, 0.31) | <0.001 |

**Notes.**

*Adjusted for age, weight, HbA1c, HDL-C, creatinine, ALT, FT4, diastolic blood pressure, smoking, drinking and exercise.

WBTOT_BMD, mean whole-body BMD; HEAD_BMD, skull BMD; LRIB_BMD, left rib BMD; RRIB_BMD, right rib BMD; T_S_BMD, thoracic spinal BMD; L_S_BMD, lumbar spinal BMD; PELV_BMD, pelvic BMD; HTOT_BMD, hip BMD; NECK_BMD, femoral neck BMD; LLEG_BMD, left leg BMD; RLEG_BMD, right leg BMD; LARM_BMD, left arm BMD; RARM_BMD, right arm BM.
