# Supplementary material for: The longitudinal associations between bone mineral density and appendicular skeletal muscle mass in Chinese community-dwelling middle aged and elderly men
Source: PeerJ. 2021 Jan 19;9:e10753. doi: 10.7717/peerj.10753 (PMC7821753; doi:10.7717/peerj.10753)
Supplement: Supplemental Information 5 [file peerj-09-10753-s005.docx]

**Table S5:**

**Associations between low lean mass changes according to ASMI and BMDs (n = 208).**

| **Outcome:*ASMI**  **（**kg/m^2^**）** | **Unadjusted**  **β coefficient (95% CI)** | | | **Adjusted***  **β coefficient (95% CI)** | | |
| --- | --- | --- | --- | --- | --- | --- |
|  | **β** | **(95% CI)** | ***P*** | **β** | **(95% CI)** | ***P*** |
| WBTOT_BMD | 1.68 | (0.81, 2.55) | <0.001 | 0.82 | (0.15, 1.48) | 0.016 |
| HEAD_BMD | -0.18 | (-0.35, -0.00) | 0.049 | -0.17 | (-0.32, -0.02) | 0.024 |
| LRIB_BMD | -0.39 | (-1.18, 0.41) | 0.346 | -0.46 | (-1.14, 0.21) | 0.178 |
| RRIB_BMD | 0.14 | (-0.28, 0.55) | 0.523 | 0.16 | (-0.21, 0.54) | 0.399 |
| T_S_BMD | 1.75 | (1.19, 2.31) | <0.001 | 0.98 | (0.51, 1.44) | <0.001 |
| L_S_BMD | 1.24 | (0.71, 1.77) | <0.001 | 0.52 | (0.10, 0.93) | 0.014 |
| PELV_BMD | 1.78 | (1.31, 2.26) | <0.001 | 0.60 | (0.21, 0.99) | 0.003 |
| HTOT_BMD | 3.44 | (2.44, 4.45) | <0.001 | 1.39 | (0.57, 2.22) | 0.001 |
| NECK_BMD | 3.44 | (2.32, 4.56) | <0.001 | 1.24 | (0.33, 2.15) | 0.008 |
| LLEG_BMD | 2.71 | (1.90, 3.52) | <0.001 | 1.20 | (0.56, 1.84) | <0.001 |
| RLEG_BMD | 2.66 | (1.87, 3.45) | <0.001 | 1.41 | (0.79, 2.03) | <0.001 |
| LARM_BMD | 3.61 | (2.23, 4.99) | <0.001 | 2.14 | (1.10, 3.19) | <0.001 |
| RARM_BMD | 4.07 | (2.83, 5.32) | <0.001 | 2.31 | (1.35, 3.28) | <0.001 |

**Notes.**

*Adjusted for age, weight, HbA1c, HDL-C, creatinine, ALT, FT4, diastolic blood pressure, smoking, drinking and exercise.

WBTOT_BMD, mean whole-body BMD; HEAD_BMD, skull BMD; LRIB_BMD, left rib BMD; RRIB_BMD, right rib BMD; T_S_BMD, thoracic spinal BMD; L_S_BMD, lumbar spinal BMD; PELV_BMD, pelvic BMD; HTOT_BMD, hip BMD; NECK_BMD, femoral neck BMD; LLEG_BMD, left leg BMD; RLEG_BMD, right leg BMD; LARM_BMD, left arm BMD; RARM_BMD, right arm BM.
