# Supplementary material for: The longitudinal associations between bone mineral density and appendicular skeletal muscle mass in Chinese community-dwelling middle aged and elderly men
Source: PeerJ. 2021 Jan 19;9:e10753. doi: 10.7717/peerj.10753 (PMC7821753; doi:10.7717/peerj.10753)
Supplement: Supplemental Information 7 [file peerj-09-10753-s007.docx]

**Table S7:**

**Associations between low lean mass changes according to ASM/BMI and BMDs (n = 208).**

| **Outcome: ASM/BMI**  **（**m^2^**）** | **Unadjusted**  **β coefficient (95% CI)** | | | **Adjusted***  **β coefficient (95% CI)** | | |
| --- | --- | --- | --- | --- | --- | --- |
|  | **β** | **(95% CI)** | ***P*** | **β** | **(95% CI)** | ***P*** |
| WBTOT_BMD | 0.05 | (-0.05, 0.16) | 0.327 | 0.04 | (-0.07, 0.15) | 0.460 |
| HEAD_BMD | -0.02 | (-0.04, -0.00) | 0.046 | -0.03 | (-0.05, -0.01) | 0.013 |
| LRIB_BMD | -0.10 | (-0.19, -0.01) | 0.035 | -0.10 | (-0.20, -0.01) | 0.028 |
| RRIB_BMD | 0.02 | (-0.03, 0.07) | 0.453 | 0.02 | (-0.03, 0.06) | 0.536 |
| T_S_BMD | 0.08 | (0.01, 0.15) | 0.017 | 0.10 | (0.03, 0.16) | 0.006 |
| L_S_BMD | 0.11 | (0.04, 0.17) | 0.001 | 0.11 | (0.04, 0.17) | 0.002 |
| PELV_BMD | 0.01 | (-0.06, 0.07) | 0.832 | 0.02 | (-0.05, 0.09) | 0.533 |
| HTOT_BMD | 0.08 | (-0.09, 0.25) | 0.348 | 0.07 | (-0.11, 0.26) | 0.443 |
| NECK_BMD | 0.12 | (-0.05, 0.30) | 0.179 | 0.12 | (-0.06, 0.31) | 0.201 |
| LLEG_BMD | 0.09 | (-0.01, 0.20) | 0.082 | 0.11 | (-0.00, 0.22) | 0.055 |
| RLEG_BMD | 0.12 | (0.02, 0.22) | 0.0191 | 0.14 | (0.04, 0.24) | 0.008 |
| LARM_BMD | 0.25 | (0.08, 0.42) | 0.005 | 0.25 | (0.08, 0.42) | 0.005 |
| RARM_BMD | 0.26 | (0.10, 0.42) | 0.001 | 0.26 | (0.10, 0.42) | 0.002 |

**Notes.**

*Adjusted for age, weight, HbA1c, HDL-C, creatinine, ALT, FT4, diastolic blood pressure, smoking, drinking and exercise.

WBTOT_BMD, mean whole-body BMD; HEAD_BMD, skull BMD; LRIB_BMD, left rib BMD; RRIB_BMD, right rib BMD; T_S_BMD, thoracic spinal BMD; L_S_BMD, lumbar spinal BMD; PELV_BMD, pelvic BMD; HTOT_BMD, hip BMD; NECK_BMD, femoral neck BMD; LLEG_BMD, left leg BMD; RLEG_BMD, right leg BMD; LARM_BMD, left arm BMD; RARM_BMD, right arm B.
